# Risk Perception, Perceived Government Coping Validity, and Individual Sleep Problems in the Early Stage of the COVID-19 Pandemic in China: Mediation Analysis Based on Negative Emotions

**DOI:** 10.3390/healthcare11070986

**Published:** 2023-03-30

**Authors:** Tao Xu, Kai Zheng, Xiaoqin Wu

**Affiliations:** 1 Department of Social Work, College of International Culture and Social Development, Zhejiang Normal University, Jinhua 321000, China; 2 Institute for Silk Road Culture and International Sinology, Zhejiang Normal University, Jinhua 321000, China; 3 Department of Economics and International Trade, School of Economics, Management & Law, Hubei Normal University, Huangshi 435002, China

**Keywords:** sleep quality, nightmares, risk perception, perceived government coping validity, COVID-19 pandemic

## Abstract

This study aimed to investigate the relationship among risk perception, negative emotions, perceived government coping validity, and the sleep problem of the public, through regression analysis and mediation analysis of data from the early stages of the COVID-19 outbreak in China (three months after the outbreak). It found that people’s perception of the risk of the pandemic, negative emotions, and perceived government coping validity significantly affected people’s sleep quality and nightmares. Further analysis found that individuals’ perception of risk not only affected their sleep but also intensified their negative emotions, ultimately impairing the quality of their sleep and leading to nightmares. However, having a high level of coping validity can mitigate negative emotions and consequently decrease the occurrence of nightmares, thereby enhancing the quality of sleep. Specifically, perceived government coping validity could not only directly reduce nightmares, but also indirectly reduce nightmares by lowering negative emotions. However, it could only indirectly improve sleep by reducing negative emotions. It implicated that improving and resolving sleep problems required not only medical intervention but also psychological intervention. Simultaneously, improving the government’s response effectiveness could strengthen people’s trust in the government, stabilize their mental states, and significantly improve their quality of life by reducing negative emotions and improving sleep.

## 1. Introduction

The COVID-19 pandemic is still ravaging the world, seriously threatening people’s health and economic recovery. COVID-19 is a kind of viral pneumonia caused by the severe acute respiratory syndrome coronavirus, characterized by strong infectivity, fast transmission speed, ease of mutation, and general susceptibility of the population [1,2]. The virus can cause serious damage to the human body, can even be life-threatening, and can result in some sequelae, such as negative emotions: anxiety, panic, and anger [3]; sleep disorders [4]; post-traumatic stress disorder (PTSD) [5]. The fear and anxiety of the virus, combined with forced lockdown and isolation procedures, caused a certain degree of sleep disorders in the general population and health workers, but the extent has not yet been fully evaluated [6]. So far, research on acute and chronic sleep disorders in COVID-19-confirmed patients is still ongoing. The present study aims to investigate the sleep problems experienced during the COVID-19 pandemic and the impact of variables such as risk perception and government response efficiency on sleep patterns. Additionally, the study seeks to explore the underlying mechanisms.

### 1.1. Sleep Problems during the COVID-19 Pandemic

A systematic review showed that the rate of insomnia symptoms rose from 20% to 45% during the COVID-19 pandemic [7]. A study from China showed that the pandemic restrictions led to sleep disorders and mental health symptoms: insomnia accounted for 29.2%, depression accounted for 27.9%, anxiety accounted for 31.6%, and acute stress accounted for 24.4% [7]. Despite some individual studies pointing out that there was no correlation between the pandemic and sleep problems [8], a large number of empirical studies showed the high prevalence of poor sleep quality and sleep disorders during the pandemic worldwide [9,10,11,12,13]. Further, many scholars have studied what factors caused sleep problems during the pandemic.

#### 1.1.1. Risk Perception and Sleep Problems

A study conducted by a three-phase neural network research group with 2363 persons found that insomnia could be predicted by various psychological factors related to the COVID-19 pandemic, including fear of infection, high intolerance to uncertainty, COVID-19-related worries, loneliness, and more severe symptoms of depression [14]. This finding supported previous research that acute infectious diseases, such as SARS, could negatively impact the psychological well-being of both infected survivors and non-infected individuals, leading to anxiety, depression, stress, and even post-traumatic stress disorder (PTSD) [15,16]. The COVID-19 pandemic, as a collective traumatic event, could generate psychological distress and anxiety symptoms, which negatively impacted sleep quality [17]. Furthermore, a recent study by Musse revealed that the frequency of nightmares increased nearly three times compared to the baseline before the pandemic, and approximately one-third of survey participants’ dream content was related to the pandemic, encompassing fears, loneliness, being chased by infected patients, and other factors [18].

The current health risks and fundamental changes in daily life during the COVID-19 pandemic might bring enormous pressure and challenges to mental health [19]. Recent studies on the general public in China found a correlation between higher rates of anxiety and depression among women, students, those with COVID-19-related symptoms, and those with a lower quality of life [20,21,22]. Another study showed that 7% of Wuhan residents reported post-traumatic stress disorder symptoms after the outbreak of COVID-19, especially among women. Meanwhile, those under 35 years old and who spent more than three hours reading COVID-19 news showed higher levels of anxiety [23]. On the other hand, several empirical studies proved that during the pandemic, the incidence of sleep disorders among the public was higher [5,14,24]. For example, a study on sleep difficulties among the Greeks by Voitsidis et al. showed that the public’s lack of awareness of possible COVID-19 exposure, intolerance of uncertainty, worries related to COVID-19, loneliness, and depression symptoms significantly increased the rates of insomnia [14].

Although the impact of the pandemic on sleep quality was still under debate and ongoing discussion, these studies almost unanimously acknowledged that the public’s concern and perception about the relationship between considering COVID-19 as a dangerous virus and the public’s sleep remained unchanged. It is worth noting that there is still a need for further research to prove whether the sleep difficulties or sleep quality of the public during the pandemic is a direct result of their understanding and perception of the public crisis. Thus, it is especially important to study the impact pathways for sleep research.

#### 1.1.2. Psychological Health and Sleep Problems

In addition to the direct impact of public perception of the risk of the virus and its danger and infectivity on sleep [25], a large number of studies showed that there was a direct or indirect impact on sleep by the emotional expression and psychological stress of the public during the pandemic. For example, negative emotions such as anxiety and depression among the public during the pandemic had a significant negative impact on sleep [26]; a study from the Netherlands found that people with good sleep quality had anxiety and other negative emotions, and the sleep quality took a turn for the worse after the outbreak of COVID-19 [14]. Undoubtedly, the significant increase in stress and burden would lead to a decrease in sleep time and sleep quality [27]; Cellini et al. verified that there were more sleep disorders for people with higher levels of depression, anxiety, and stress symptoms: the longer the sleep time was, the lower the sleep quality was [28]. A cross-sectional study from Morocco confirmed that during the COVID-19 pandemic, the incidence of sleep disorders, anxiety, and depression was higher, and sleep quality decreased significantly; it also confirmed that an incorrect understanding of sleep could cause negative impacts such as anxiety and depression, and these negative impacts affected sleep and caused sleep disorders and reduced sleep quality [12].

Emotions had an impact on sleep regardless of population and region. A UK study found that anxiety and depression symptoms in elderly people were significantly related to sleep quality, with those without anxiety or stress having better sleep quality [29]. On the contrary, elderly people with moderate to severe anxiety, compared to elderly people with mild anxiety, were more likely to have symptoms of insomnia during isolation [30]. Similarly, Mishra et al. [31] found through studying the psychological health of elderly people during the pandemic that those with stress, anxiety, and depression had worse sleep quality and were more likely to have sleep disorders such as insomnia. A study on the psychological reactions of Wuhan citizens to COVID-19 also found that their mental health and sleep quality were relatively worse compared to before the pandemic, and medical staff was more likely to feel anxious [32]. These studies indicated that during the COVID-19 pandemic, there was a significant correlation between changes in negative emotions and sleep quality, with an increase in negative emotions leading to or exacerbating the decline in sleep quality.

#### 1.1.3. Perceived Government Coping Validity and Sleep Problems

The current research on the quality of sleep and sleep disorders among the public during the COVID-19 pandemic mostly called on the government to improve public mental health, and to prevent the spread of the virus and its impact on society or individual body health, as seen in the studies of Brooks, Lakhan, Barros Marilisa, etc. [17,33,34]. Aside from some studies exploring the mediating role of government on the impact of psychological stress on sleep [35], fewer scholars focused on the mechanisms of the government’s response to the impact of sleep. In China, whether the government could respond promptly to public requests, could accurately and fully disclose information, and could properly handle these matters were not only related to political stability and the construction of a harmonious society but also easily became an important criterion for the public’s evaluation of government behavior [36]. Only when the public believed that the government should be responsible for the occurrence of sudden public events would the government’s credibility be questioned. Currently, from the perspective of the factors affecting the public’s sleep during the COVID-19 pandemic, there was little research on the impact of the government’s response to the public’s sleep.

### 1.2. New Analytical Framework and Hypothesis

The cognitive-mediational theory of emotions suggested that the level of stress caused by a stressor was determined by an individual’s thoughts and appraisals of it [37,38]. According to this theory, there were two steps to determine the level of threat or harm caused by a stressor and one’s response to it. First, an individual estimated the severity of the stressor and classified it as either a threat or a challenge (primary appraisal). If the stressor was perceived as a threat, then secondary appraisal followed, along with physical and emotional reactions. In this step (second), one evaluated the available resources to deal with the stressor, such as time, money, and physical ability (secondary appraisal). Insufficient resources to meet the demands of the environment could lead to stress, which could then change one’s emotional state and affect their health [39,40,41]. This theory provided insight into the impact of the current pandemic (stressor) on emotional reactions (perceived stress) and bodily responses (sleep quality).

**Figure 1:**
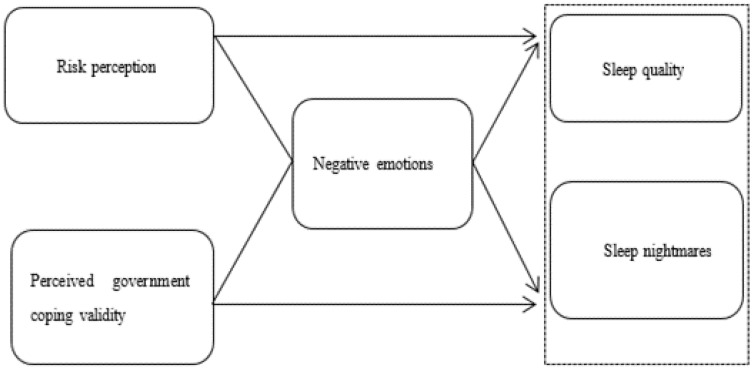
A conceptualized analytical framework.

The cognitive-mediational model, although it examined the impact of an individual’s cognition and psychological feedback on their activities, neglected the impact of the environment in which the individual was. Yet, an individual’s activities were always in a certain natural and social environment and were surely influenced by the environment [42]. It meant that the individual’s cognition and mediation would be affected by the environment and reflected feedback. Therefore, we developed a comprehensive analytical framework “Cognitive-mediational-reflection Model” for the analysis of sleep problems during the COVID-19 pandemic (Figure 1).

Previous studies explored and confirmed that public perception of COVID-19, including their own risk and its consequences, significantly affected their emotions and mental health, potentially leading to negative emotions such as anxiety and depression [19,23,43,44]. Additionally, a large number of studies showed that public concern and worries about COVID-19 might affect sleep quality, causing nightmares, awakenings, and other problems [10,11,34,35,45,46]. Based on this, we logically infer that the public’s risk perception of COVID-19 can directly or indirectly affect sleep through negative emotions such as anxiety and worries.

Therefore, we propose Hypothesis 1: *Public risk perception and negative emotions have a significant impact on sleep quality and nightmares, and negative emotions play a significant mediating role between public risk perception and sleep quality and nightmares.*

Additionally, previous research has already indicated that human behavior was influenced by cognition and environmental factors—individual personality traits, cognitive biases, external environment, and their interaction [47] Studies showed that during the COVID-19 pandemic, trust in the government could increase the public’s confidence in controlling the pandemic, reduce negative emotions, and improve sleep quality [48,49]. The degree to which the public had confidence in the government was largely based on the government’s effectiveness in handling the pandemic [50,51,52,53]. Based on this, it was reasonable to speculate that the government’s effectiveness might enhance public confidence, reduce psychological stress, mitigate the psychological impact, and thereby alleviate sleep problems and reduce the likelihood of nightmares.

Therefore, we propose research Hypothesis 2: *The public’s evaluation of the government’s response behavior and negative emotions have a significant impact on their sleep quality and nightmares, and negative emotions play an important mediating role between the public’s evaluation of the government’s response behavior and sleep quality and nightmares.*

## 2. Data and Measurement

### 2.1. Data

After the outbreak of the COVID-19 pandemic, some researchers at the university where the author is affiliated initiated a study project on the risk perceptions and social consequences of COVID-19. The research project aimed to understand people’s perceptions of the early outbreak of COVID-19 and its potential consequences. In the initial design stage of the project, a pilot survey was conducted on a small scale, and the questionnaire was subsequently revised again based on relevant issues identified through the pilot survey. This formal survey was conducted online in February 2020 three months after the outbreak of the COVID-19 pandemic. The survey was administered to more participants through the snowball method by WeChat and QQ. A total of 1613 questionnaires were collected, covering 30 provinces, municipalities, and autonomous regions in China. After removing 76 incomplete surveys where more than one-third of the questions were left unanswered, the analysis was based on 1537 responses. Although some of the initial participants were known to the researchers, the demographic data collected through the snowball sampling method were largely consistent with current data, except for some specific population variables, making it representative to some extent [25,54]. The respondents completed the questionnaire without any financial incentives, which definitely resulted in more realistic data. Despite the limitations of online questionnaires, they still held practical significance considering the COVID-19 outbreak situation.

### 2.2. Measures

#### 2.2.1. Dependent Variables

This study primarily explored sleep problems, which were measured by two dimensions: subjective sleep quality evaluation and assessment of repeated nightmares during sleep. The questionnaire mainly measured these two aspects through questions on poor sleep quality and frequent nightmares. The alternative answers were “completely not conform”, “somewhat not conform”, “Neutral”, “somewhat conform”, and “completely conform”, and it was coded as 1–5.

#### 2.2.2. Independent Variables

*Risk Perception of COVID-19.* To measure the “Risk Perception of COVID-19”, an independent variable, we used a set of subjective evaluation items as per previous studies [55,56,57,58]. The first three items evaluated the level of infection, fatalities from COVID-19, and the level of severity of the illness, using a five-point ordinal scale (strongly disagree, disagree, neutral, agree, strongly agree), which was coded as 1 to 5. The next three items gauged the likelihood of the virus affecting oneself, friends and family, and the general public, using a five-point scale (not likely to very likely) also coded as 1 to 5. The last item measured the level of worry about the virus, with answers ranging from not worried at all to very worried in 5 equal intervals, coded as 1 to 5. The reliability of this scale was established through a Cronbach’s alpha score of 0.72. The scores of all items were summed to create an index of the risk perception of COVID-19, drawing on previous studies.

*Negative emotions.* Western psychologists defined emotion as a complex pattern of physical and psychological changes, including physiological arousal, sensations, cognitive processes, and behavioral responses [59]. Chinese scholars defined emotion as a psychological activity centered on an individual’s desires and needs, an individual’s attitude experience, and the corresponding behavioral response to objective things [56]. Emotion psychologists believed that emotion was a multi-component, multi-dimensional-structure, multi-level-integration, and psychological-activity process and psychological motivational force that interacted with cognition for the survival adaptation of organisms and interpersonal communication [58]. Due to a lack of consensus on the concept of emotion, the definition of emotional communication was also difficult to express accurately.

In this study, we defined negative emotions as individual emotions, specifically referring to the negative emotions you feel in the face of COVID-19 including doubt, tension, worry, helplessness, panic, sadness, and fear. The questionnaire included a set of subjective evaluation variables that scale psychological emotions, including doubt, tension, worry, helplessness, trepidation, sadness, and fear, with answers ranging from very inconsistent to very consistent in 5 intervals. Through aggregation, we attained the dependent variable. Specifically, we first tested the correlation of the emotional variables’ indicators and found that the indicators were closely related. The test result of reliability was α = 0.9035, and the Kaiser–Meyer–Olkin value was 0.8738 (*p* < 0.000), indicating that these indicators were suitable for exploratory factor analysis. Therefore, we conducted exploratory factor analysis by principal components and varimax rotation. From the result of Supplementary Table S1 and Figure S2, we could see that only the eigenvalue of factor 1 was larger than 1, which showed that only one factor could be extracted. So, we extracted the factor and called it negative emotions according to the correlation of indicators and their meanings. The factor score was saved as the independent variable.

*Perceived government coping validity.* It was assessed as the public’s subjective assessment of the speed and efficiency of measures taken by the government during the pandemic. To study its impact on government credibility during the COVID-19 pandemic, a scholar divided it into four components: proactivity, responsiveness, transparency, and accuracy [60]. However, when evaluating the government’s response measures during the pandemic, how to divide the dimensions was quite complex. To overcome this, exploratory factor analysis was conducted to extract the factors that represented the government’s evaluation of its response to public crises, or the government’s response validity. This approach proved to be reliable and valid, with a reliability score of α = 0.829 and KMO = 0.83 (*p* < 0.001) (details can be found in the Supplementary Table S3 and Figure S4).

#### 2.2.3. Control Variables

*Gender* was a binary variable (coded as 0 for male and 1 for female). *Education level* was an ordinal variable (elementary and below, junior high, high school, university, Master’s degree or above). *Age* was measured as a ratio variable, and the square of age was created as a control variable. *Marital status* was a categorical variable, including married and unmarried, with married being the reference group.

### 2.3. Models

In this study, descriptive statistics and regression analysis were performed. The cross-tabulation of nominal variables such as gender, marital status, census registration, and individual’s sleep quality and nightmares was described. The correlations among individual risk perception, perceived government coping validity, negative emotion, and individual sleep quality and nightmares were also analyzed.

Six ologit models were then set-up to examine the effect of risk perception and perceived government coping validity on individual sleep quality and nightmares. The models were constructed through the F-test and the coefficients were calculated with the *t*-test, with significant results marked by asterisks.

First, we conducted a cross-tabulation description on nominal variables such as different genders and regions, different risk perceptions, and individual sleep quality and nightmares. At the same time, we also described and analyzed the correlation between individual risk perception consequences, perceived government coping validity, and individual sleep quality and nightmares.

Secondly, we set-up three different logit models to explore how the core independent variables such as risk perception, perceived government coping validity, and negative emotions affected individual sleep quality and nightmares. All the models built were through the F-test, and the coefficients in the models were calculated through the *t*-test, with the significance marked by an asterisk.

Thirdly, to explore the mechanisms of the impacts of individual risk perception and perceived government coping validity on individual sleep quality and nightmares, we built a model with negative emotion as the mediating variable and explored their different mechanisms.

As the explanatory variables in this paper were ordinal measured variables, the independent variables were gradually included in the model to explore the links between the independent variables and several different dependent variables. We, therefore, used the generalized ordered logistic regression model described by Williams [61] with the following model setup:

(1) $$\mathrm{Pr}(Y_{I}>j)=\frac{\exp\left(\alpha_{j}+X_{i}\beta_{j}\right)}{1-\left[\exp\left(\alpha_{j}+X_{i}\beta_{j}\right)\right]}\;,\;j=1,2,3,4,5$$

In this model, we set “j” to transform the multi-classification problem into a classification problem with classification objectives of {1…j} and {j + 1…k}. The logit defined based on these two classes represented the logarithm of the cumulative probability of belonging to the k-j classes concerning the cumulative probability of the previous “j” classes, called the cumulative dominance model. Therefore, in this analysis, the dependent variable Y took values from 1 to 5, so the model was as follows:

$$P_{1}=P\left(y=1|x\right)=\frac{\exp\left(\alpha_{1}+\beta_{x}\right)}{1+\exp\left(\alpha_{1}+\beta_{x}\right)}\;\;p_{1}=p\left(y=1\right)=p_{1}$$

$$P_{2}=P\left(y=2|x\right)=\frac{\exp\left(\alpha_{2}+\beta_{x}\right)}{1+\exp\left(\alpha_{2}+\beta_{x}\right)}\;\;p_{2}=p\left(y=2\right)=p_{2}-p_{1}$$

……

$$P_{5}=P\left(y\leq 5|x\right)=1{\;\;p}_{5}=p\left(y=5\right)=1-p_{4}$$

The first model represented the relationship between the probability P of the dependent variable y taking the first value and x, and the second model represented the relationship between the cumulative probability P of y taking the first two values and x. These models had different constant terms and identical regression coefficients. $p\left(1\right)=p_{1}$ was for the probability of y taking the first value, $p\left(2\right)=p_{2}-p_{1}$ was for the probability of y taking the second value, and $p\left(5\right)=1-p_{4}$ was for the probability of y taking the fifth value. The analyses in this paper were based on Stata 16.0.

## 3. Results

### 3.1. Descriptive Analysis

The descriptive statistics and correlation analysis of the main variables are shown in Table 1. In terms of sample proportion, the sleep quality score of female students was 2.68 and that of male students was 2.63, both with a nightmare score of 2.16. Chi-square analysis showed that gender was not related to sleep quality and nightmares (*p* > 0.05). From the perspective of age, the analysis objects were generally younger, and the correlation analysis results showed that age was correlated with sleep quality (*p* < 0.05) but not with a nightmare (*p* > 0.05). In terms of education, the proportion of this group with higher education was higher, and the average education was higher. The correlation analysis showed that the two variables were not related in terms of education (*p* > 0.05), and nightmares were not associated with marital status (*p* > 0.05), but sleep quality was related to marital status (*p* < 0.001). However, the correlation results of the census register with the dependent variable were opposite to those of marital status; risk perception, negative emotions, and evaluations of the government’s response effectiveness were all significantly related to sleep quality and nightmares during the pandemic (*p* < 0.05) (see Table 1).

### 3.2. Regression Results

In order to explore the relationship between risk perception, negative emotion, government coping validity, and sleep quality, we, respectively, constructed models 1a, 1b, and 1c, followed by models 2a, 2b, and 2c, to explore the relationship between risk perception, negative emotions, government coping validity, and sleep quality and having nightmares.

To analyze the impact of people’s risk perception on public sleep quality and nightmares in the early stage of the pandemic, we established models 1a and 2a (Table 2). The results of Model 1a indicated that the regression coefficient of people’s risk perception on sleep quality was 0.217 (*p* < 0.001), which meant that in the same case of other predictive variables, for every 1-unit increase in the risk perception of the Chinese public, the probability of sleep quality deterioration would increase by 24.23% (e^0.217^−1). It could be seen that the results of the model indicated a positive prediction relationship between the risk perception of the early stage of the pandemic and the poor sleep quality of the Chinese public.

The research results of Model 2a also showed that the regression coefficient of people’s risk perception of nightmares was 0.3 (*p* < 0.001), which meant that in the same case of other predictive variables, for every 1-unit increase in the risk perception of the Chinese public, the probability of having nightmares could increase by 34.99% (e^0.3^−1). Combining the results of Models 1a and 2a, we could conclude that the stronger the risk perception of the public, the worse the sleep quality and the easier it was to have nightmares during sleep.

To analyze the impact of the negative emotions of the public on their sleep quality and nightmares during the early stage of the pandemic, we established models 1b and 2b (Table 2). As seen from Model 1b: the regression coefficient of negative emotions of the public on sleep quality was 0.79 (*p* < 0.001), indicating that, with other predictive variables being the same, the probability of sleep quality deterioration increased by 1.2 times (e^0.79^−1) for every 1-unit increase in negative emotions of the public. Thus, the negative emotions of the public toward the pandemic had a positive impact on their sleep quality; Model 2b showed that the regression coefficient of negative emotions of the public on nightmares was 0.783 (*p* < 0.001), indicating that, with other predictive variables being the same, the probability of sleep quality deterioration increased by 1.19 times (e^0.783^−1) for every 1-unit increase in negative emotions of the public. Hence, there was a close positive relationship between the public’s negative emotions and sleep quality deterioration and nightmares.

Finally, to analyze the relationship between the public’s evaluation of the government’s response actions and sleep quality during the pandemic, we established models 1c and 2c. Model 1c showed that the regression coefficient between the public’s evaluation of the government’s response to the pandemic and their sleep quality was −0.095 (*p* < 0.05), indicating that, with other predictive variables being the same, the probability of sleep quality deterioration decreased by 9.1% (1−e^−0.095^) for every 1-unit increase in the public’s evaluation of the government’s response actions, and there was no significant relationship between sleep quality deterioration and public’s evaluation; Model 2c showed that the regression coefficient between the public’s evaluation of the government’s response actions and nightmares was −0.122 (*p* < 0.001), indicating that, with other predictive variables being the same, the probability of nightmares decreased by 11.5% (1−e^−0.122^) for every 1-unit increase in the public’s evaluation of the government’s response actions, and there was a close negative relationship between the public’s evaluation of the government’s response actions and nightmares.

### 3.3. Mediation Analysis

The above analysis discussed the impact of risk perception, perceived government coping validity, and negative emotions on sleep. To further reveal their impact path and mechanism on sleep, two different mediating models were constructed. In these two models, negative emotions acted as mediating variables between risk perception, perceived government coping validity, and their impact on sleep quality and nightmares.

From Table 3, negative emotions had a significant mediating effect on the relationship between the public’s risk perception and sleep quality, with a mediating effect ratio of 72.53%. It could be seen that negative emotions had a positive mediating effect between the public’s risk perception of COVID-19 and sleep quality. Similarly, negative emotions also had a significant mediating effect on the relationship between the public’s risk perception and sleep nightmares, with a mediating effect ratio of 50.56%. This indicated a significant mediating effect between the public’s risk perception of COVID-19 and sleep nightmares, and the indirect effect c’ in the mediating effect was positive, which meant that the public’s risk perception was indirectly influenced by negative emotions and further exacerbated their sleep quality and sleep nightmares during the pandemic. Therefore, negative emotions not only had a direct effect on sleep quality and sleep nightmares in the early stages of the pandemic but also played an important mediating role between them. As a result, research hypothesis H1 has been fully verified.

However, in terms of the relationship between the perceived government coping validity and negative emotions and sleep nightmares, the indirect effect was significant, which meant that there was a significant mediating effect between negative emotions and perceived government coping validity and sleep nightmares, with a mediating effect ratio of 24.57%. Unlike the pattern of negative emotions’ influence on the public’s risk perception and sleep, the indirect effect c’ in this model was negative, which meant that a positive evaluation of the government’s response would reduce negative emotions and thus reduce sleep nightmares. The indirect effect of negative emotions between the perceived government coping validity and sleep nightmares was significant, as well as the direct effect, which meant that the perceived government coping validity had both a significant direct and indirect effect on sleep nightmares through negative emotions. As for sleep quality, the perceived government coping validity indirectly improved the sleep quality by reducing negative emotions while not directly improving them (Table 4). Hence, hypothesis H2 was partially confirmed.

## 4. Discussion

This study analyzed online survey data from the early stage of the COVID-19 pandemic to explore the impacts and pathways between people’s risk perception and perceived government coping validity on sleep. The study found that:

To start with, the public’s perception of COVID-19 risk affected not only their sleep quality but also the frequency of nightmares. The higher the perceived risk, the worse the sleep quality and the higher the probability of nightmares. This was consistent with previous research on the impact of fear and risk perception of COVID-19 on sleep [5,12,14,15,24,27]. Compared to previous research, one of the contributions of this study was that we not only re-verified the impact of risk perception on sleep quality and nightmares but also revealed that risk perception directly led to poor sleep quality, increased the likelihood of nightmares, and caused negative emotions, which indirectly affected sleep quality and nightmares. This conclusion has not been found in previous research.

Furthermore, this study also found a significant association between government response behavior and sleep quality and nightmares. Although previous research found that people’s trust in the government and evaluation of government response behavior significantly affected the efficiency and consequences of combating COVID-19 [53,62], the relationship between government response behavior and sleep quality has not been studied. This study revealed that a positive evaluation of government response behavior significantly improved sleep quality and reduced the probability of nightmares, but the mechanisms of action were different. For nightmares, government response behavior not only directly reduced the probability of nightmares but also reduced negative emotions by improving sleep quality.

Once again, negative emotions have been revealed to be associated with sleep quality and nightmares. This finding is consistent with previous research conclusions that negative emotions such as anxiety and worry related to COVID-19 could worsen people’s sleep quality [5,13,24,26,33,35]. However, the mechanisms behind this relationship have not been thoroughly explored. The current study found that negative emotions not only directly affected sleep quality and nightmares, but also acted as the mediating variable that impacted them. Specifically, negative emotions exacerbated poor sleep quality and increased the likelihood of having nightmares, but also mediated the relationship between sleep quality and nightmares through their effect as a mediating variable for perceived risk and perceived government coping validity. Negative emotions enhanced the negative impact on sleep quality and the likelihood of nightmares through their role as a mediating variable for perceived risk, but perceived government coping validity reduced negative emotions, thus mitigating the negative impact on sleep quality and nightmares.

In conclusion, this study made an important contribution by revealing the two distinct mechanisms: sleep quality and nightmares were both affected by risk perception and perceived government coping validity; they were influenced by negative emotions, and the difference was that risk perception positively exacerbated sleep problems but perceived government coping validity negatively mitigated sleep problems.

The above conclusions have important implications for how different countries should address the COVID-19 pandemic. First, COVID-19 not only caused measures to be taken to provide medical assistance but also had a significant negative impact on individual mental health, which in turn affected their normal life in the long term [4,30,44,63]. Thus, governments around the world should not only respond from a medical and public health perspective but also pay attention to changes in people’s mental states and provide necessary psychological help and intervention. Secondly, sleep problems may be an important feature of the long-term impact of COVID-19 [64,65]. Improving and resolving sleep problems required not only medical intervention but also psychological intervention [43,66]. Increasing the efficiency of response to COVID-19 by governments could strengthen people’s trust in the government and stabilize their mental states, thereby significantly reducing negative emotions, improving sleep quality, and enhancing the quality of life.

This study also had some limitations. In the first step, the data were collected over a short period through an online survey. Due to the inherent overall unclear and ambiguous boundaries of the online survey method, the representativeness of the study may have some issues. In the second step, the sample in this study was imbalanced in terms of gender ratios and had a higher level of educational attainment among participants, which had some impact on the conclusions of the study, but these two indicators were not core explanatory variables and had a limited impact on the conclusions of the study. In the third step, the research object was only the Chinese public two months after the early outbreak of COVID-19, and this paper only focused on the impact of the public’s risk perception, negative emotions, and the effectiveness of the government’s response on individual sleep during the early stage of the pandemic. As time passed, the overreactions of risk perception would tend to be normal, and with the development and spread of the pandemic, the virus’s mutation could cause emotional changes, and the government’s response and various policies also had undergone significant changes. These factors need to be studied in-depth in subsequent research.

## 5. Conclusions

First, the public’s risk perception and negative emotions both had a significant negative impact on the quality of sleep and sleep nightmares during the early stage of COVID-19: the stronger the risk perception and the more frequent the negative emotions, the worse the quality of sleep and the more prone the public was to nightmares. Second, the public’s risk perception not only directly affected the quality of sleep and sleep nightmares, but also intensified the public’s negative emotions toward pandemic prevention, thereby indirectly affecting the quality of sleep and sleep nightmares. Third, perceived government coping validity affected the quality of sleep as well as sleep nightmares; simultaneously, negative emotions mediated the effect of government response validity on sleep nightmares and sleep quality. Among them, the impact of perceived government coping validity on sleep quality was completely mediated by negative emotions, and the impact on nightmares was partially mediated.

## Figures and Tables

**Table 1:** Descriptive Results: tabulation and correlation.

Variables		Sleep Quality (SQ)		Sleep Nightmares (SN)	
		Mean	Sig.	Mean	Sig
Sex	Male	2.63	*p* > 0.05	2.16	*p* > 0.05
	Female	2.68		2.16	
Marital status	Married	2.58	*p* < 0.001	2.15	*p* > 0.05
	Single	2.87		2.19	
Census register	Rural	2.70	*p* > 0.05	2.67	*p* < 0.01
	Urban	2.64		2.08	
		Coef.	Sig.	Coef.	Sig.
Age		0.136	*p* < 0.001	0.032	*p* > 0.05
Education		−0.038	*p* > 0.05	−0.032	*p* > 0.05
Risk perception		0.132	*p* < 0.001	0.133	*p* < 0.001
Negative emotions		0.903	*p* < 0.001	0.292	*p* < 0.001
Perceived government coping validity		0.883	*p* > 0.05	−0.032	*p* > 0.05

**Table 2:** Ologit regression model results.

	Sleep Quality (SQ)			Sleep Nightmares (SN)		
Variables	Model 1a	Model 1b	Model 1c	Model 2a	Model 2b	Model 2c
Sex (Female = 0)	0.127	−0.002	−0.001	0.042	−0.105	−0.125
	[0.206]	[0.982]	[0.993]	[0.684]	[0.325]	[0.242]
Age	0.064 *	0.034	0.035	0.022	−0.019	−0.024
	[0.094]	[0.373]	[0.369]	[0.588]	[0.637]	[0.563]
Age2	−0.000	−0.000	−0.000	−0.000	0.000	0.000
	[0.347]	[0.890]	[0.887]	[0.798]	[0.410]	[0.375]
Education	−0.025	−0.035	−0.036	−0.005	−0.008	−0.002
	[0.317]	[0.155]	[0.153]	[0.847]	[0.766]	[0.934]
Marital status (Married = 0)	−0.145	−0.024	−0.024	−0.202	−0.119	−0.119
	[0.455]	[0.903]	[0.903]	[0.310]	[0.560]	[0.560]
Census register (Rural = 0)	−0.165 *	−0.126	−0.128	−0.345 ***	−0.311 ***	−0.287 ***
	[0.095]	[0.207]	[0.203]	[0.001]	[0.003]	[0.006]
Risk perception (RP)	0.217 ***	0.115 **	0.114 **	0.300 ***	0.225 ***	0.231 ***
	[0.000]	[0.041]	[0.042]	[0.000]	[0.000]	[0.000]
Negative emotions (NM)		0.790 ***	0.787 ***		0.783 ***	0.816 ***
		[0.000]	[0.000]		[0.000]	[0.000]
Perceived government coping validity (PGCV)			−0.095 * [0.0301]			−0.122 ** [0.016]
cut1	−0.077	−0.846	−0.845	−0.114	−0.860	−0.858
	[0.892]	[0.142]	[0.142]	[0.847]	[0.158]	[0.159]
cut2	0.194	−0.554	−0.552	0.221	−0.495	−0.492
	[0.733]	[0.336]	[0.337]	[0.709]	[0.417]	[0.419]
cut3	2.005 ***	1.422 **	1.424 **	2.506 ***	1.943 ***	1.952 ***
	[0.000]	[0.014]	[0.014]	[0.000]	[0.001]	[0.001]
cut4	4.062 ***	3.598 ***	3.600 ***	4.358 ***	3.853 ***	3.864 ***
	[0.000]	[0.000]	[0.000]	[0.000]	[0.000]	[0.000]
N	1611	1611	1611	1611	1611	1611
Pseudo-R2	0.0129	0.0590	0.0590	0.0114	0.0598	0.0613

*** *p* < 0.001, ** *p* < 0.01, * *p* < 0.05.

**Table 3:** Mediation effects of negative emotions.

		Coef	Std Err	Z	P > Z	LLCI	ULCI	$\left(c/\hat{c^{\prime }}\right)$
RP—NM—SQ	Indirect effect	0.061	0.012	4.969	0.000	0.377	0.501	72.53%
	Direct effect	0.084	0.033	2.537	0.011	0.019	0.150	
RP—NM—SN	Indirect effect	0.056	0.011	4.964	0.000	0.346	0.461	50.56%
	Direct effect	0.111	0.031	3.609	0.000	0.051	0.172	
PGCV—NM—SN	Indirect effect	−0.087	0.011	−7.71	0.000	−0.109	−0.065	24.57%
	Direct effect	0.077	0.026	2.91	0.004	0.025	0.130	
PGCV—NM—SQ	Indirect effect	−0.107	0.0134	−7.98	0.000	−0.134	−0.081	100%
	Direct effect	0.045	0.030	1.49	0.136	−0.014	0.103	

**Table 4:** Main results of models.

Variables	Sleep Quality	Nightmares
Risk Perception (RP)	Direct/Indirect	Direct/Indirect
Perceived government coping validity (PGCV)	Indirect	Direct/Indirect

## Data Availability

The data presented in this study are available on request from the corresponding author.

## Supplementary Materials

The following supporting information can be downloaded at: https://www.mdpi.com/article/10.3390/healthcare11070986/s1, Table S1. Exploratory factor analysis of panic; Figure S1. Scree plot of eigenvalues after factor analysis; Table S2: Perceived government coping validity factor loadings table and gravel plot; Figure S2: Eigenvalue gravel plot of perceived government coping validity.
