# Supplementary material for: Risk Perception, Perceived Government Coping Validity, and Individual Sleep Problems in the Early Stage of the COVID-19 Pandemic in China: Mediation Analysis Based on Negative Emotions
Source: Healthcare (Basel). 2023 Mar 30;11(7):986. doi: 10.3390/healthcare11070986 (PMC10094412; doi:10.3390/healthcare11070986)
Supplement: Supplementary file 1 [file healthcare-11-00986-s001.zip › healthcare-2243198-supplementary.pdf]

Table S1. Exploratory factor analysis of panic.

Variable	Factor1	Factor2	Factor3	Uniqueness
doubt	0.5361	0.18	0.0583	0.6768
tension	0.7968	0.3068	-0.0672	0.2665
worry	0.7484	0.3641	0.0134	0.3071
helplessness	0.8019	-0.167	0.0163	0.3287
trepidation	0.8625	-0.2397	-0.059	0.1952
sadness	0.7211	-0.1222	0.1048	0.4541
fear	0.842	-0.2193	-0.0303	0.242
Eigenvalue	4.09839	0.40747	0.02375	
Cumulative variance	0.9773	0.9844	0.9861	

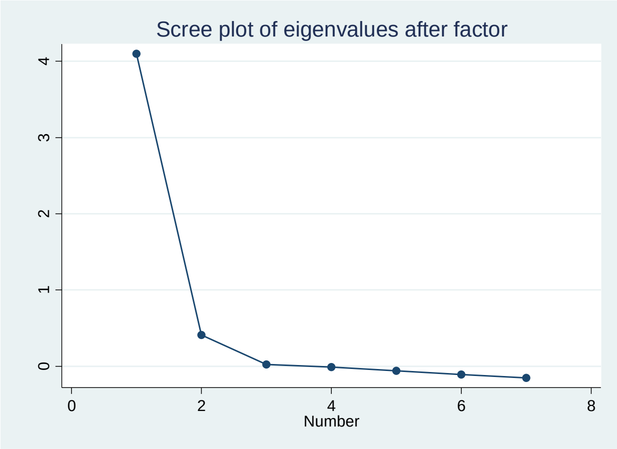

Figure S1. Scree plot of eigenvalues after factor.

Table S2: Perceived government coping validity factor loadings table and gravel plot

Variable Name	Mean	Std. Dev.	Factor
Citizens' satisfaction with the series of measures taken by the government in coping with Covid-19	4.04	1.331	0.839
Citizens' approval of the central government's current actions in response to Covid-19	4.40	1.149	0.799
Citizens' approval of local government's current actions in response to Covid-19	4.09	1.374	0.797
Citizens' evaluation of the effectiveness of measures implemented by the Hubei Provincial Government to contain the spread of Covid-19	3.75	1.092	0.675
The extent to which citizens trust their local government to control local outbreaks effectively	4.60	1.115	0.738
Eigenvalue			2.98
Contribution rate %			59.55
KMO			0.827
Alpha			0.829

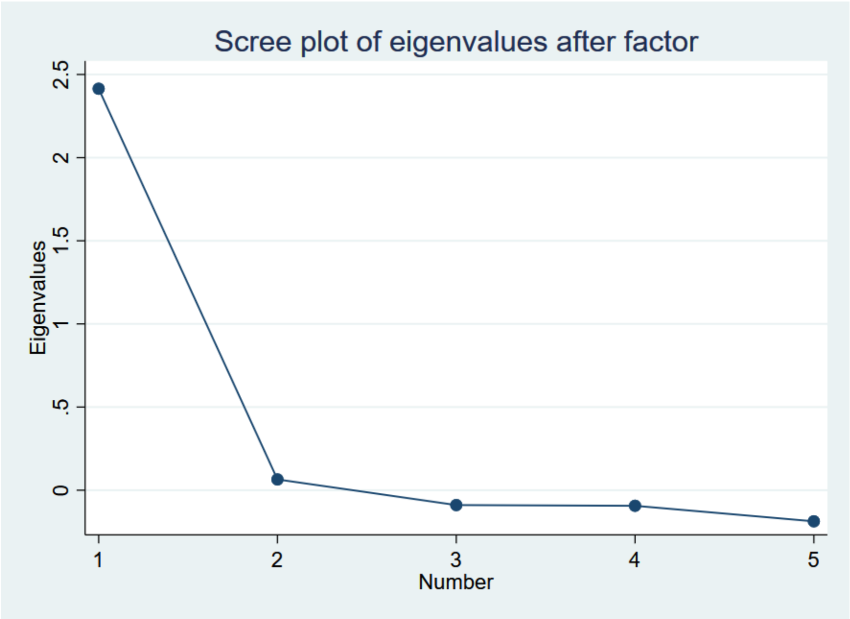

Figure S2: Eigenvalue gravel plot of perceived government coping validity
